# Supplementary material for: Metabolomics Signatures and Subsequent Maternal Health among Mothers with a Congenital Heart Defect-Affected Pregnancy
Source: Metabolites. 2022 Jan 21;12(2):100. doi: 10.3390/metabo12020100 (PMC8877777; doi:10.3390/metabo12020100)
Supplement: Supplementary file 1 [file metabolites-12-00100-s001.zip › metabolites-1495397-supplementary.pdf]

Supplemental Figures & Tables

Supplemental Table S1. Metabolites and the LODs quantified by compound class from the AbsoluteIDQ® p400 HR Kit in the study.

Compound Class	# of Metabolites in the Compound Class	Average LOD (µM)	Range (µM)
Amino acids	21	0.9 ± 0.87	0.05–3.76
Biogenic amines	21	0.031 ± 0.56	0.01–2.48
Monosaccharide	1	51	NA
Acylcarnitines	55	0.13 ± 0.7	0.01–5.32
Diglycerides	18	0.44 ± 0.54	0.06–1.93
Triglycerides	42	0.36 ± 0.43	0.03–1.96
Lysophosphatidylcholinesas	24	0.64 ± 1.7	0.01–8.38
Phosphatidylcholines	172	0.27 ± 0.82	0.01–8.76
Sphingomyelins	31	0.11 ± 0.15	0.01–0.81
Ceramides	9	0.07 ± 0.03	0.02–0.12
Cholesteryl esters	14	3.44 ± 2.56	0.04–8.93

Supplemental Table S2. Summary of Data Processing Results.

ID	Features (Positive)	Imputed (<LOD)	Features (Processed)
S34	353	47	400
S73	384	16	400
S7	353	47	400
S14	383	17	400
S19	356	44	400
S56	347	53	400
S33	348	52	400
S2	351	49	400
S28	353	47	400
S57	360	40	400
S44	357	43	400
S13	353	47	400
S82	376	24	400
S23	359	41	400
S58	355	45	400
S29	347	53	400
S70	378	22	400
S54	356	44	400
S77	380	20	400
S68	380	20	400
S43	378	22	400

ID	Features (Positive)	Imputed (<LOD)	Features (Processed)
S24	382	18	400
S81	381	19	400
S5	355	45	400
S78	381	19	400
S4	360	40	400
S1	380	20	400
S63	386	14	400
S17	348	52	400
S72	376	24	400
S32	377	23	400
S39	382	18	400
S31	349	51	400
S75	384	16	400
S48	346	54	400
S47	362	38	400
S27	352	48	400
S11	350	50	400
S52	343	57	400
S60	381	19	400
S15	362	38	400
S37	358	42	400
S40	358	42	400
S76	356	44	400
S51	352	48	400
S8	385	15	400
S25	350	50	400
S80	385	15	400
S38	348	52	400
S12	350	50	400
S45	354	46	400
S62	345	55	400
S41	340	60	400
S6	382	18	400
S9	353	47	400
S22	382	18	400
S61	357	43	400
S59	358	42	400
S50	348	52	400
S49	357	43	400
S74	384	16	400
S10	384	16	400

ID	Features (Positive)	Imputed (<LOD)	Features (Processed)
S36	382	18	400
S71	384	16	400
S79	356	44	400
S55	355	45	400
S16	386	14	400
S67	378	22	400
S64	353	47	400
S30	348	52	400
S35	347	53	400
S21	357	43	400
S65	352	48	400
S66	383	17	400
S53	357	43	400
S46	385	15	400
S20	380	20	400
S42	358	42	400
S18	355	45	400
S69	353	47	400
S26	381	19	400
S3	341	59	400

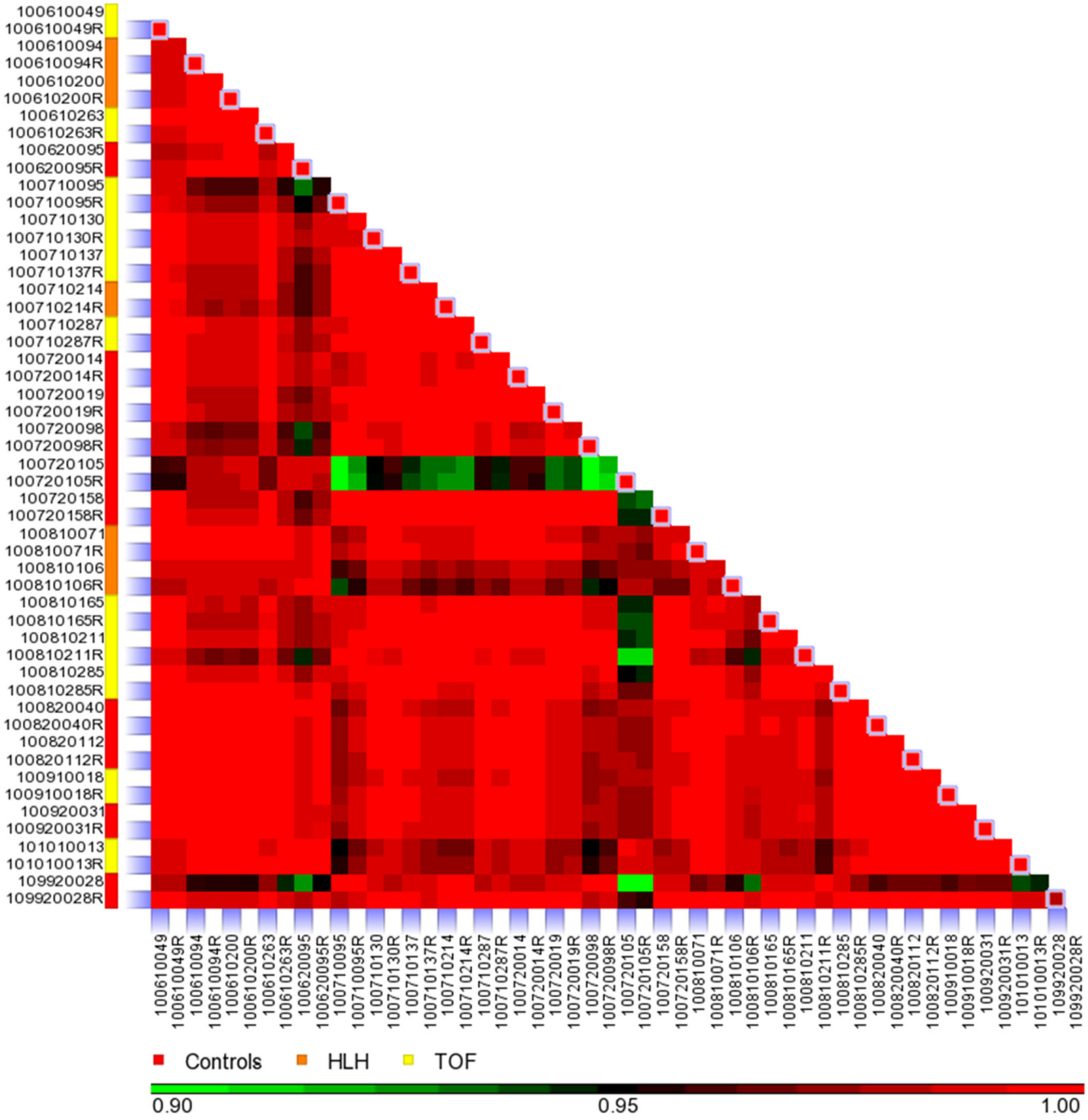

Supplemental Figure S1. Correlation matrix represents the reproducibility of targeted metabolomics data among technical repeats. Boxes highlighted in blue represent the correlation between samples and its repeats on the metabolite intensity detected. Relative correlation was represented using a red–black–green color scale, with red represents high correlation and green indicates low correlation.

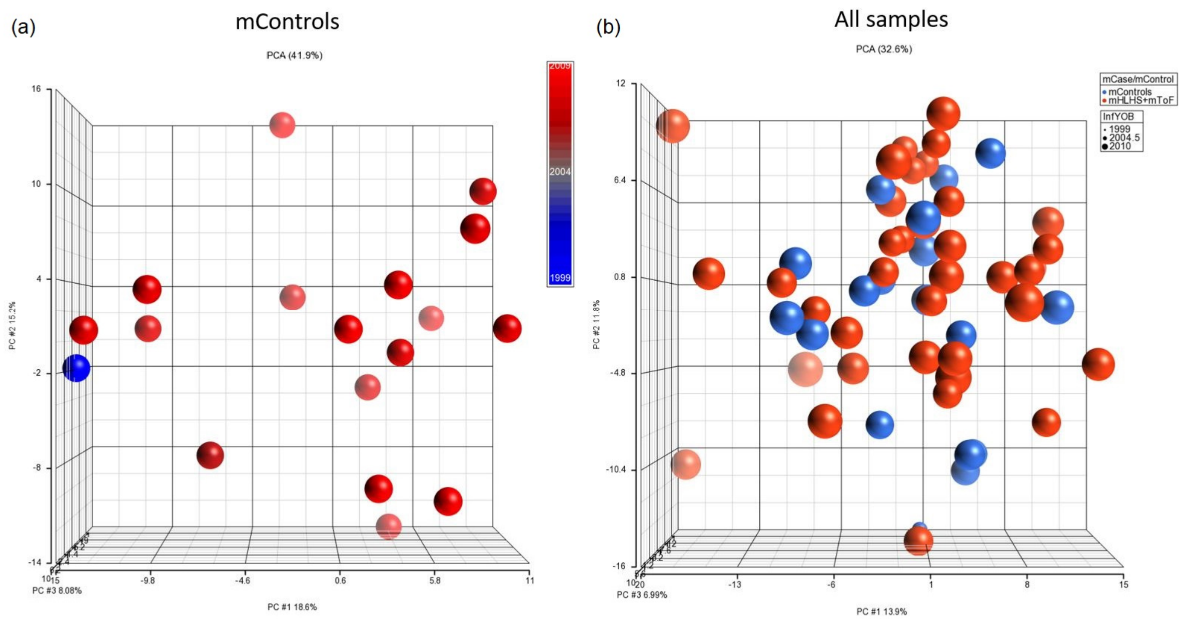

Supplemental Figure S2. Principle component analysis of (a) mControl samples, colored by the infant year of birth; and (b) all samples including mControl (blue dots), mHLHS and mToF (red dots). The size of dots representing the infant year of birth.

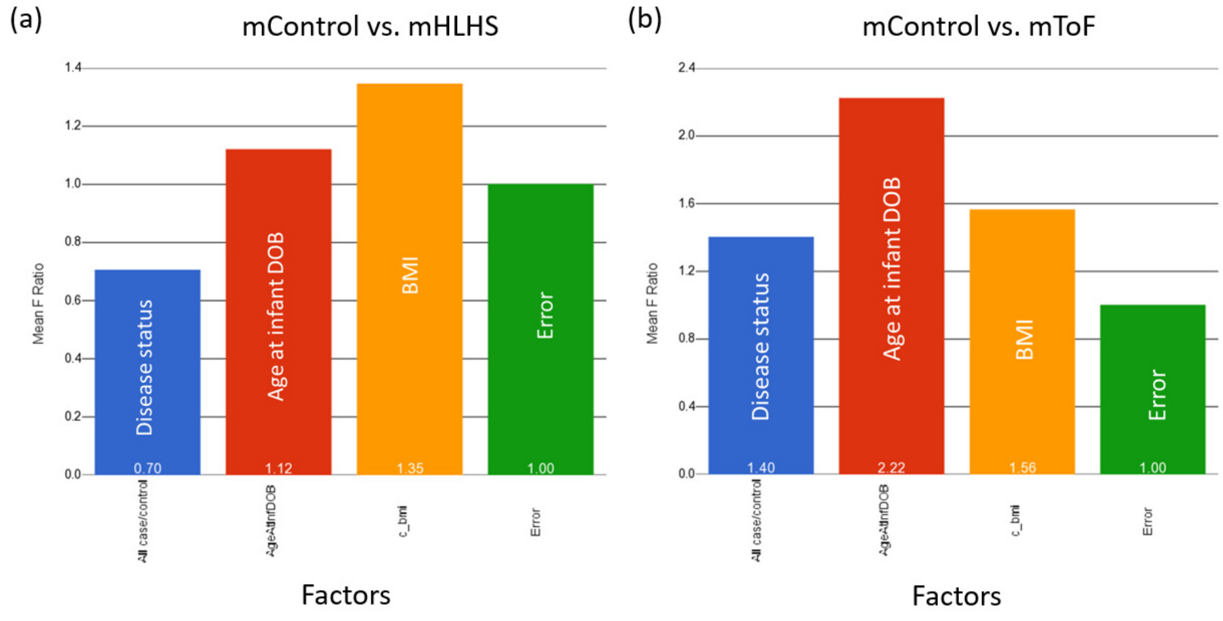

Supplemental Figure S3. Sources of variations plots. Factors (maternal age at delivery and BMI) in the ANCOVA model are listed on the x-axis (including random error). The y-axis represents the Mean F-ratio (variability in the data not explained by the other factors) of all the metabolites.
